# Proteomic exploration reveals a metabolic rerouting due to low oxygen during controlled germination of malting barley (*Hordeum vulgare* L.)

**DOI:** 10.3389/fpls.2023.1305381

**Published:** 2023-12-11

**Authors:** Clare E. O'Lone, Angéla Juhász, Mitchell Nye-Wood, Hugh Dunn, David Moody, Jean-Philippe Ral, Michelle L. Colgrave

**Affiliations:** ^1^ Australian Research Council Centre of Excellence for Innovations in Peptide and Protein Science, Edith Cowan University, School of Science, Joondalup, WA, Australia; ^2^ Commonwealth Scientific and Industrial Research Organization, Agriculture and Food, ACT, Canberra, ACT, Australia; ^3^ Pilot Malting Australia, Edith Cowan University, School of Science, Joondalup, WA, Australia; ^4^ Barley Breeding, InterGrain Pty Ltd, Bibra Lake, WA, Australia; ^5^ Commonwealth Scientific and Industrial Research Organization, Agriculture and Food, Brisbane, QLD, Australia

**Keywords:** barley, malt, controlled germination, malting, low-oxygen, proteomics, mass spectrometry, plant breeding

## Abstract

Barley (*Hordeum vulgare* L.) is used in malt production for brewing applications. Barley malting involves a process of controlled germination that modifies the grain by activating enzymes to solubilize starch and proteins for brewing. Initially, the grain is submerged in water to raise grain moisture, requiring large volumes of water. Achieving grain modification at reduced moisture levels can contribute to the sustainability of malting practices. This study combined proteomics, bioinformatics, and biochemical phenotypic analysis of two malting barley genotypes with observed differences in water uptake and modification efficiency. We sought to reveal the molecular mechanisms at play during controlled germination and explore the roles of protein groups at 24 h intervals across the first 72 h. Overall, 3,485 protein groups were identified with 793 significant differentially abundant (DAP) within and between genotypes, involved in various biological processes, including protein synthesis, carbohydrate metabolism, and hydrolysis. Functional integration into metabolic pathways, such as glycolysis, pyruvate, starch and sucrose metabolism, revealed a metabolic rerouting due to low oxygen enforced by submergence during controlled germination. This SWATH-MS study provides a comprehensive proteome reference, delivering new insights into the molecular mechanisms underlying the impacts of low oxygen during controlled germination. It is concluded that continued efficient modification of malting barley subjected to submergence is largely due to the capacity to reroute energy to maintain vital processes, particularly protein synthesis.

## Introduction

Barley (*Hordeum vulgare* L.) is an economically important cereal crop in world malt production. Raw barley grain is converted to malt in a process of controlled germination called malting. Malting involves physical and biochemical changes to obtain fermentable products necessary for brewing. The quality attributes of malted barley are a critical factor in the brewing process, affecting the color, flavor, and overall beer quality. The Kolbach index (KI) is a key indicator of the level of enzymatic activity or protein hydrolysis during the malting process, with desirable levels around 35.0-49.9% (Fox et al., 2003). Genomic diversity and breeding have contributed to producing quality malt from barley grown worldwide, where efficient water uptake and timely uniform germination are pivotal determinants of malt quality (Chandra et al., 1999; Briggs, 2002; MacLeod and Evans, 2016).

Controlled germination or malting is a three-stage process that begins with water uptake (imbibition) during steeping, where the raw grain is submerged in water, raising grain moisture to above 42%. Although there are periods of air rest, the immersed grain experiences phases of low-oxygen (hypoxia <21% O_2_) resulting from reduced air diffusion (Gibbs and Greenway, 2003). This oxygen deficiency leads to impaired mitochondrial activity. It triggers a switch from aerobic to anaerobic respiration, rapidly reducing cellular adenosine triphosphate (ATP) production (Bailey-Serres and Voesenek, 2008; Magneschi and Perata, 2009). Aerobic respiration is returned in the second stage, germination, where the imbibed grain is incubated for 4-5 days in an aerated (normoxic >21% O_2_), moist atmosphere with rotation. This is the most metabolically active stage, and enzymes such as proteases, amylases, hemicellulases, and oxidases are involved in the continued embryo growth and formation of green malt. In the final stage, kilning, the green malt is heat treated, reducing the moisture to around 12% and decreasing enzymatic activity. Followed by a second drying step to stop all biological processes, reducing moisture contents to 4-5% and stabilizing the final characteristics of the malt (Briggs et al., 2004; Daneri-Castro et al., 2016a). Overall, malting is a resource-intensive process, and one critical area of concern is reducing water usage with efforts to maintain modification efficiency crucial for the industry’s long-term sustainability (Daneri-Castro et al., 2016b; Izydorczyk and Edney, 2017). By exploring the molecular mechanisms of controlled germination, we can gain insights into implementing reduced water usage while maintaining malt quality, which is an attractive proposition in an internationally competitive market.

Proteomics, the large-scale study of the protein complement of a cell, tissue, or organism under a specific, defined set of conditions, is a powerful approach for investigating the changes in protein abundance that occur during barley germination. The barley proteome and metabolome have been widely studied due to their importance in agriculture and the malting and brewing industry, recently reviewed by Bahmani, O’Lone (Bahmani et al., 2021), Fox and Watson-Fox (2021), and Fox and Bettenhausen (2023). In the present study, we used quantitative proteomics via the sequential window acquisition of all known theoretical spectra-mass spectrometry (SWATH-MS) (Gillet et al., 2012) to quantify proteins across the malting time course in a newly developed breeding line, IGB1467, that demonstrated efficient proteolysis at a lower moisture content than a traditional malting barley cultivar, Flinders. In early stages of controlled germination, aerobic respiration is inhibited due to an O_2_ deficiency caused by submergence, and despite the grain’s sensitivity to low-O_2_ conditions, the stress can be managed for a limited time by inducing an adaptive response in protein synthesis and changes to carbohydrate metabolism. Previous studies have revealed that in low-O_2_ environments, a metabolic shift occurs to increase the anaerobic production of ATP via cytosolic glycolysis, influencing the mobilization of carbohydrates to promote substrate-level ATP production (Lasanthi-Kudahettige et al., 2007; Bailey-Serres et al., 2012). The objective of this study was to evaluate the proteomic and physiochemical changes that occur at 24-hour intervals during the first 72 hours of malting, where the grain experiences submergence stress. We aimed to identify key protein groups responsible for the observed differences in malting phenotypes under low-O_2_ stress to help improve our understanding of their molecular roles. Our study highlights metabolic patterns of proteins and enzymes involved in signaling and regulating mRNAs associated with core metabolic responses, including reconfiguring the carbohydrate metabolism to develop a more efficient grain modification while maintaining malt quality for sustainable malting practices.

## Materials and methods

### Plant material, malting conditions, and malt analysis

Malt barley (*Hordeum vulgare* L.) breeding line IGB1467 (IGB; experimental) and its’ parental breeding line, cultivar Flinders (FLN; control), were grown at an InterGrain (Bibra Lake, Western Australia) experimental field site, Brookton, Western Australia (32°18’05’ S, 117°14’32’ E). Grain evaluation data is provided in **Supplementary T** **able S1**. These two closely related barley genotypes were selected based on demonstrated phenotypic trait differences in water uptake, malting potential, and malt characteristics from a previous project carried out at Pilot Malting Australia (PMA, Edith Cowan University, Australia (**Supplementary** **Figure S1**).

Grains (100 kg) were pilot malted at PMA (**Supplementary Tables S2**, **S3**) and sampled at approximately 0, 24, 48, and 72 hours after imbibition (HAI). The four sampling times corresponded to key stages along the malting time course: (1) 0 HAI, raw barley grain; (2) 24 HAI, exit steep, following periods of grain submergence (with aeration) and air rest; (3) 48 HAI, germination, stage 1 and (4) 72 HAI germination, stage 2. Pilot malting at PMA closely aligns with industrial-scale malting sample sizes in comparison to micro- and benchtop-malting but was limited in that it did not allow for directly comparable malting run samples. Each genotype was malted to meet accepted malt quality parameters (**Supplementary T** **able S4**), specifically a similar KI between 37-47%. The KI was calculated using Equation 1.


$$\text{Equation }1:\;Kolbach\;Index\;\left(\%\right)=\left(\frac{Soluble\;Protein}{Protein\;Content}\right)\times 100$$

For each time point, 20 g of grain with rootlet maintained (removed for malt quality analysis), was collected, snap-frozen, and stored at -80°C until processing. All grains were thawed and inspected to exclude contamination and freeze-dried for 72 h to remove all moisture. The grains were milled using a Retsch Mixer Mill MM 400 (Metrohm, NSW, AUS) and sieved using a 300 µm sieve (Endecotts Pty Ltd. Sieves, London, UK) to produce a fine grade wholemeal flour.

### Protein extraction and digestion

Protein was extracted from wholemeal flour without defatting (20 mg, n = 4) of malting barley grain at each time point using 400 µL (20 µL/mg) of 8 M urea and 2% (w/v) dithiothreitol (DTT) buffer. The suspension was vortexed and sonicated (Soniclean Ultrasonic Cleaner 25HD, 650W, 43 kHz) for 5 min at room temperature. The samples were incubated on a shaker block (Thermo-Scientific, AUS) at 400 rpm for 45 min at room temperature (RT). The solutions were centrifuged for 15 min at 20,800 x g, and the protein extracts (supernatant) were used for subsequent reduction, alkylation, and filter-aided (MWCO 10kDa) digestion using trypsin (Promega, NSW, AUS) as per established methods (Colgrave et al., 2016). Protein concentrations were determined via a Varioscan plate reader (Thermo-Scientific, AUS) using Bradford protein assay (California, USA) following the manufacturer’s protocol with dilutions and BSA standard curve. The tryptic peptides were re-suspended in ddH_2_O containing 1% formic acid with the addition of iRT reference peptide solution (1 pmol/μL; Biognosys, Zurich, CHE) for subsequent LC-MS/MS analysis.

### Sequential window acquisition of theoretical mass spectra (SWATH-MS) data acquisition

The peptide fractions (1 μL) were separated through reverse-phase chromatography Ekspert nanoLC415 (Eksigent, Dublin, CA, USA) and analyzed online in a TripleTOF 6600 mass spectrometer (SCIEX, Redwood City, CA, USA) as described previously (Bose et al., 2020). In brief, peptides were desalted by loading on a ChromXP C18 (12 nm, 3 μm, 120 Å, 10 × 0.3 mm) trap column at a flow rate of 10 μL/min and separated on a ChromXP C18 (12 nm, 3 μm, 120 Å, 150 × 0.3 mm) column at a flow rate of 5 μL/min. The mobile phase A (0.1% formic acid with 5% dimethyl sulfoxide, DMSO in 94.9% ddH_2_O) and mobile phase B (0.1% formic acid with 5% DMSO in 90% acetonitrile and 4.9% ddH_2_O) were used to establish a linear gradient composed of 68 min of 3-25% B, 5 min 25-35% B with an increase to 80% B for 2 min, and a 2 min hold at 90% B, return to 3% B over 1 min, followed by re-equilibrating at 3% B for 8 min. The flow rate was 0.3 µL/min, and the elute from the HPLC was directly coupled to the DuoSpray source of the TripleTOF 6600 MS (SCIEX). The ion spray voltage was set to 5,500 V; the curtain gas was set to 138 kPa (20 psi), and the ion source gas 1 (GS1) and gas 2 (GS2) were set to 103 and 138 kPa (15 and 20 psi). The heater interface was set to 150°C. The TOF–MS survey scan was collected over the mass range of *m/z* 360–2000 with a 250 ms accumulation time, and the product ion mass spectra were acquired over the mass range of *m/z* 150–1800 with a 30 ms accumulation time using rolling collision energy and a collision energy spread (CES) of 5. Variable window SWATH acquisition was employed using 30 SWATH windows (including 1 Da overlap) spanning the mass range of *m/z* 150–2000 with SWATH windows determined using the SWATH Variable Window Calculator 1.0 (SCIEX) for a total cycle time of 1.15 s.

### Protein identification and quantitative data processing

SWATH-MS files were processed in DIA-NN ver.1.8 using deep neural networks (DNNs) inference algorithm (Demichev et al., 2020). The spectral output was searched against *in silico* tryptic digests of *Hordeum vulgare* subset of the UniProt-KB database (ver.2022/10; 54,629 sequences) appended, with Biognosys iRT pseudo-protein sequence and the common Repository of Advantageous Proteins database (cRAP) (Frankenfield et al., 2022). DIA-NN quantitative analysis was performed using tryptic peptides of 7 to 30 amino acids in length, with up to one missed cleavage. As a fixed modification, carbamidomethylation of cysteine was selected, and no variable modifications were allowed. The precursor *m/z* range, 300-1800, was selected, and the fragment ion m/z range was 200-1800. The algorithm settings included the automatic modes for mass accuracy, MS1 accuracy, and scan window with and removing likely interferences the software predicted. The neural network classifier was run in single-pass mode with a high-accuracy quantification strategy, and cross-run normalization was performed in a retention time-dependent manner. Peptides were identified after applying a 1% False Discovery Rate (FDR). Identifications from the cRAP database were ignored.

### Bioinformatics and statistical analysis

Protein sequences of interest were BLAST searched against the Morex genome ver.2^1^ using CLC Main Workbench (QIAGEN; ver.22.0.2). Protein Gene Ontology term annotations were predicted using eggNOG-mapper^2^, Gene Ontology (GO) database^3^, and UniProt mapping database^4^ (all databases accessed 2023/03). Additional GO annotation was taken from the previously published barley genome annotation files (Mascher et al., 2017) and cross-referenced with the Barley Reference Transcript dataset (BaRT) downloaded (2022/10) from the Barley Expression Database (EoRNA)^5^ GO annotation for the BaRT gene IDs dataset was done using the available spreadsheet^6^. Functional pathway analysis was performed using the Kyoto Encyclopedia of Genes and Genomes (KEGG) mapper and reconstruct software tools^7^ to annotate protein KEGG ortholog numbers and corresponding functional distributions (**Supplementary Data S1**).

Unsupervised principal component analysis (PCA) was conducted to identify possible outliers resulting from technical (processing and instrumental) procedures and to evaluate groupings within the data set. The identification of differentially abundant proteins (DAPs) within genotype over the time course was carried out using DESeq2 analysis (Love et al., 2014) of the following pairwise comparisons 24/0, 48/24, and 72/48 HAI (log_2_ Fold Change (log_2_ FC) ≥1.5, *p ≤*0.05). Parametric Analysis of Gene Set Enrichment (PGSEA; *p ≤*0.05, Gene set size min. 5, max. 2000) (Kim and Volsky, 2005; Furge et al., 2012) with GO enrichment analysis (biological process) was performed using iDEP.96 Shiny R platform^8^. Hierarchical Cluster Analysis (HCA; one minus Pearson correlation with complete linkage, cluster cut-off height 0.65) was performed on the 50 topmost significant pathways (*p* =<1.0e-13) using the Morpheus^9^ web tool. The mean relative abundance of log_2_ transformed data (n=4) was visualized using Morpheus^9^ and integrated into pathway diagrams using BioRender^10^. The compositional statistical analyses were carried out using R ver.3.6.1^11^ in RStudio ver.2023.09.1+494^12^.

### Measurement of physiochemical parameters

The physiochemical parameters of malting barley grain were measured at approximately 0, 24, 48, and 72 HAI, using 10 mg of wholemeal flour in triplicates for total starch, expressed as percentage dry weight; free glucose and ethanol-soluble carbohydrate expressed in milligrams per gram wholemeal; and total α-amylase activity expressed in ceralpha units (CU) per gram wholemeal. Total starch was measured using a Total Starch Assay Kit (Megazyme, Neogen Australasia, AUS) and modified as described by Zhang, Pritchard (Zhang et al., 2022). Absorbance was read at 510 nm after a 20-minute incubation at 50°C using a SPECTROstar Nano Microplate Reader (BMG LABTECH, AUS). A standard curve was generated using kit-supplied glucose at 1 mg/ml concentration. Total soluble sugar and free glucose were extracted as described by Zhang, Pritchard (Zhang et al., 2022), and all ethanol-soluble fractions were pooled. Total soluble sugar was measured using anthrone reagent (0.2% anthrone, in 70% H_2_SO_4_ v/v) and glucose at 1 mg/mL concentration standard curve (Whan et al., 2014). Free glucose was determined following the method described by Campbell, Hansen (Campbell et al., 1999). Total α-amylase activity was measured using a α-Amylase Assay Kit (Megazyme) adapted for flat-bottom 96-well microplate, following the manufacturer’s protocol using SPECTROstar Nano Microplate Reader (BMG LABTECH).

## Results

In this study, a comprehensive proteome-wide approach was employed to explore differences between two malting barley genotypes, (i) IGB; experimental breeding line, and (ii) FLN; control cultivar, that malted to meet malt quality specification (**Supplementary T** **able S4**). While the two genotypes took different treatment times in the steep to meet these specifications, they were malted to a similar KI, IGB, 45%, and FLN, 43%, a key parameter indicative of the level of grain proteolysis. The aim was to dissect the proteome of IGB and FLN to identify key protein groups and explore differences in abundance patterns in the complex metabolic processes affected by water uptake and modification efficiency differences in the two genotypes at each time point.

### Protein identification and profiling of malting barley during controlled germination

SWATH-MS processing identified, quantified, and annotated 3,485 protein groups (**Supplementary Data S1**). Unsupervised preliminary data exploration via principal component analysis (PCA) was applied to characterize the samples. Along PC1, significant variation (33.6%) was seen due to the time point, while PC2 (13.0%) separated the samples based on the genotype (**Supplementary Figure S2**). In IGB and FLN, 3,193 and 3,142, respectively, out of the 3,485 proteins detected were seen in all four time points. The results show that the proteomes were significantly altered after imbibition and during controlled germination, with clear separations between genotypes and the four-time points.

### Differentially abundant protein (DAP) identification and exploration of malting barley during controlled germination

#### DAP identification and pairwise analysis

**Figure 1:**
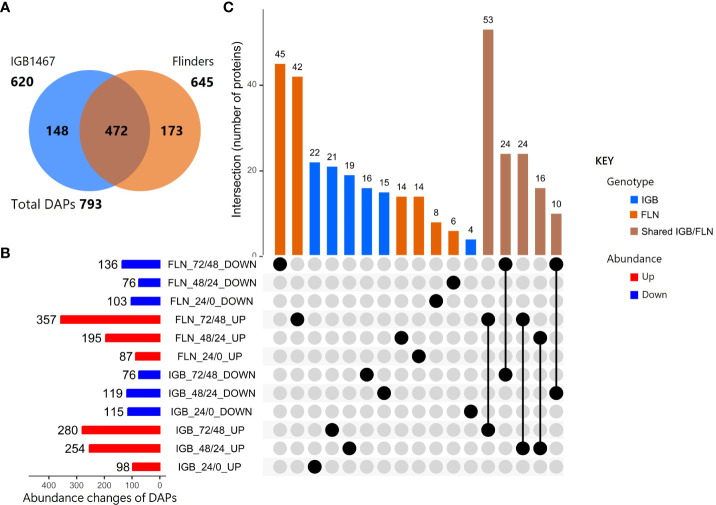
Differentially abundant protein (DAP) groups (log_2_ FC ≥1.5, *p ≤*0.05) in two malting barley genotypes comparing time points at 0, 24, 48, and 72 HAI. **(A)** Venn diagram of DAPs observed uniquely or commonly (overlap) in IGB1467 and Flinders. **(B)** UpSet Plot with bar graph and matrix representing the up-and down-regulation of the DAPs with comparisons across time points. **(C)** Column graph illustrating the shared intersections in the matrix of DAPs between genotype and time points.

**Figure 2:**
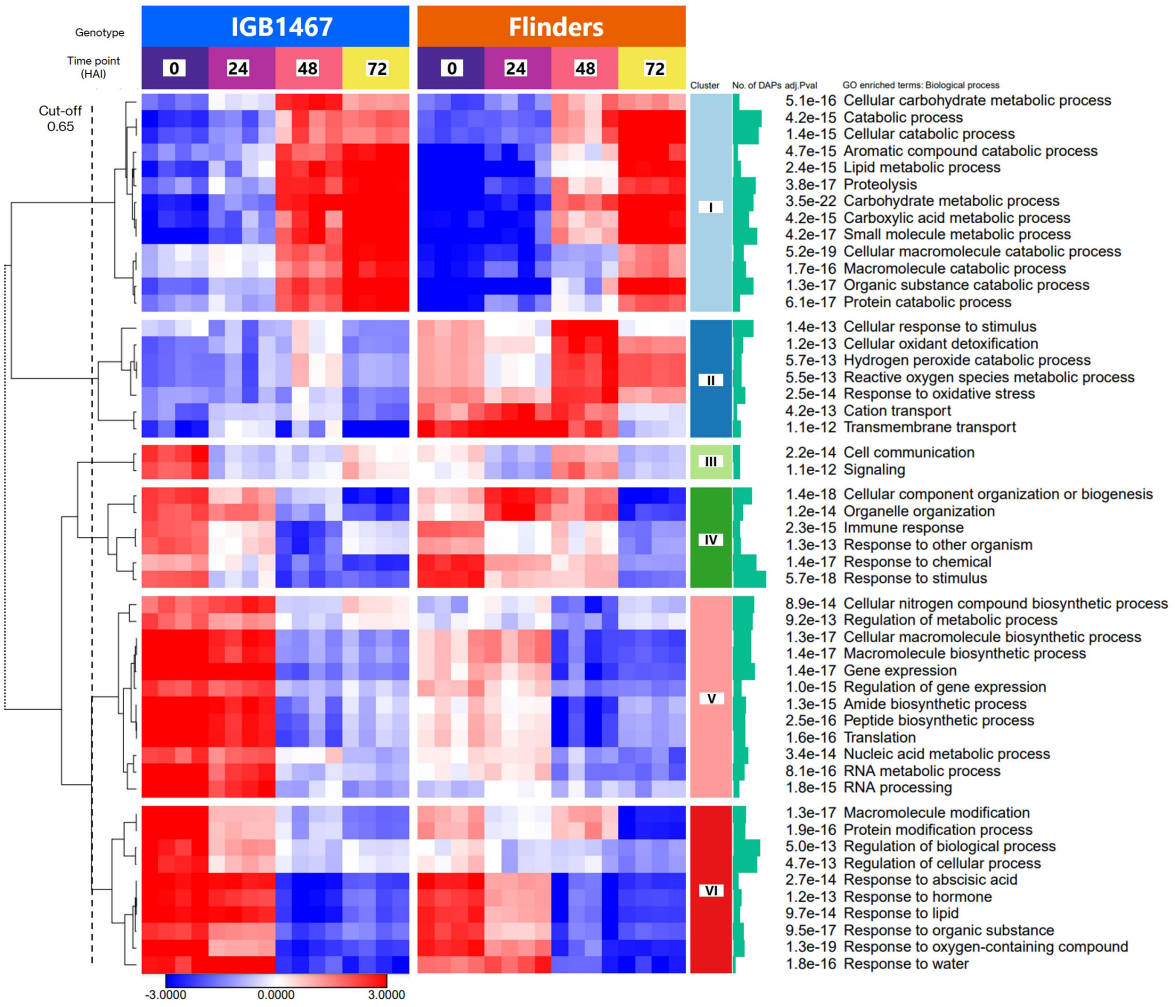
Parametric analysis of gene set enrichment (PGSEA, with all DAPs, min. gene set size, 5; *p ≤*0.05), with hierarchical clustering analysis (HCA; cut-off 0.65, Pearson correlation, complete linkage) of the top 50 significant gene ontology (GO) enriched biological process pathways for the identified differentially abundant protein groups (log_2_ FC≥1.5, *p ≤*0.05).

In the six pairwise comparisons, 793 DAPs were identified and presented in multiple comparisons (**Supplementary Data S2**). Of the DAPs, 620 and 645 were found in IGB and FLN, of which 472 were shared, and 148 or 173 were uniquely present in either IGB or FLN, respectively (**Figure 1A**). The DAPs of IGB and FLN and their overlapping relationships and abundance changes (increase/decrease) across the controlled germination time course are shown in the UpSet analyses (**Figure 1B**; **Supplementary Data S3**). The DAPs with decreased abundance shared a similar pattern across both genotypes and controlled germination time points. However, DAPs with increased abundance had varied patterns between genotypes. In IGB, the DAP increase was similar at 48/24 HAI (254) and 72/48 (280). However, in FLN, the greatest increase was observed at 72/48 HAI with 357 DAPs. Most DAPs shared between IGB and FLN were identified at 72/48 HAI for both increased (53) and decreased (24) DAPs (**Figure 2C**). The subsequent most significant alteration was noted in the comparison at 48/24 HAI (IGB) and 72/48 HAI (FLN) with 24 DAPs. (**Figure 1C**).

#### Gene set enrichment analysis highlights DAPs involved in controlled germination of malting barley

To illustrate the significant difference in the biological response to controlled germination, identified DAPs from IGB and FLN across all time points were analyzed via clustering of the PGSEA results.. The 50 topmost significant pathways (*p* ≤1.0e-13) were clustered into six distinct accumulation patterns (**Figure 2**, cluster cut-off height = 0.65, **Supplementary Data S3**). The six clusters identified are explained here briefly with detailed data and discussion shown in the supplementary document (**Supplementary Table** **S5**).

Overall, cluster I (**Figure 2**; light blue) highlighted increased protein abundance at 48 and 72 HAI. This cluster is primarily enriched in proteins involved in the ‘Cellular macromolecule catabolic process’ (*p*=5.2e-19), ‘Catabolic process’ (*p*=4.2e-15) and ‘Proteolysis’ (*p*=3.8e-17). Although both genotypes shared a similar pattern, IGB demonstrated an earlier increase in abundance, with many changes occurring by 48 HAI seen in FLN at 72 HAI. Cluster II (**Figure 2**; dark blue) demonstrated to be significantly greater abundance in FLN, across all time points. This cluster included GO terms related to ‘Transmembrane transport’ (*p*=1.1e-12)’, ‘Response to oxidative stress’ (*p*=2.5e-14), and ‘Hydrogen peroxide catabolic process’ (*p*=5.7e-13). Cluster III (**Figure 2**; light green) highlighted greater protein abundance at 0 HAI in IGB, while in FLN, the greatest abundance is present at 48 HAI. The two GO terms in this cluster are related to ‘Signaling’ (*p*=1.1e-12) and ‘Cell communication’ (*p*=2.2e-14).

Clusters IV (**Figure 2**; dark green) were related to ‘Response to stimulus’ (*p*=5.7e-18), with shared abundance patterns at 0 and 24 HAI between the genotypes. However, IGB abundance decreased earlier decrease at 48 HAI, as seen in FLN at 72 HAI. Cluster V (**Figure 2**; pink) demonstrated significantly greater abundance in IGB and was related to GO terms, ‘Translation’ (*p*=1.6e-16), ‘Peptide biosynthetic process’ (*p*=2.5e-16), and ‘RNA metabolic process’ (*p*=8.1e-15), all linked to protein synthesis. Finally, cluster VI (**Figure 2**; red) terms highlighted the greatest abundance in both genotypes in the early stages at 0 and 24 HAI, decreasing over the time course. This cluster was enriched with proteins involved in ‘Regulation of biological process’ (*p*=5.0e-13) and ‘Protein modification’ (*p*=1.9e-16). DAP abundance varied significantly amongst Clusters I, II, and V, with the most significantly enriched GO term ‘Carbohydrate metabolic process’ (*p*=3.5e-22).

Functional proteome analysis reveals metabolic rerouting due to low oxygen during controlled germination Pathways-associated analysis found that of the 793 DAPs, 55.9% (443) were successfully mapped to 20 KEGG functional categories (**Supplementary Figure S4**; **Supplementary Data S1**), with 20.0% (89) of proteins involved in carbohydrate metabolism, followed by genetic information processing, making up a further 14.4% (64) and biosynthesis of other secondary metabolites contributing 7.0% (31) to the protein classification. Similar to the findings in the PGSEA, the highest number of DAPs were found to be associated with carbohydrate metabolism.

**Figure 3:**
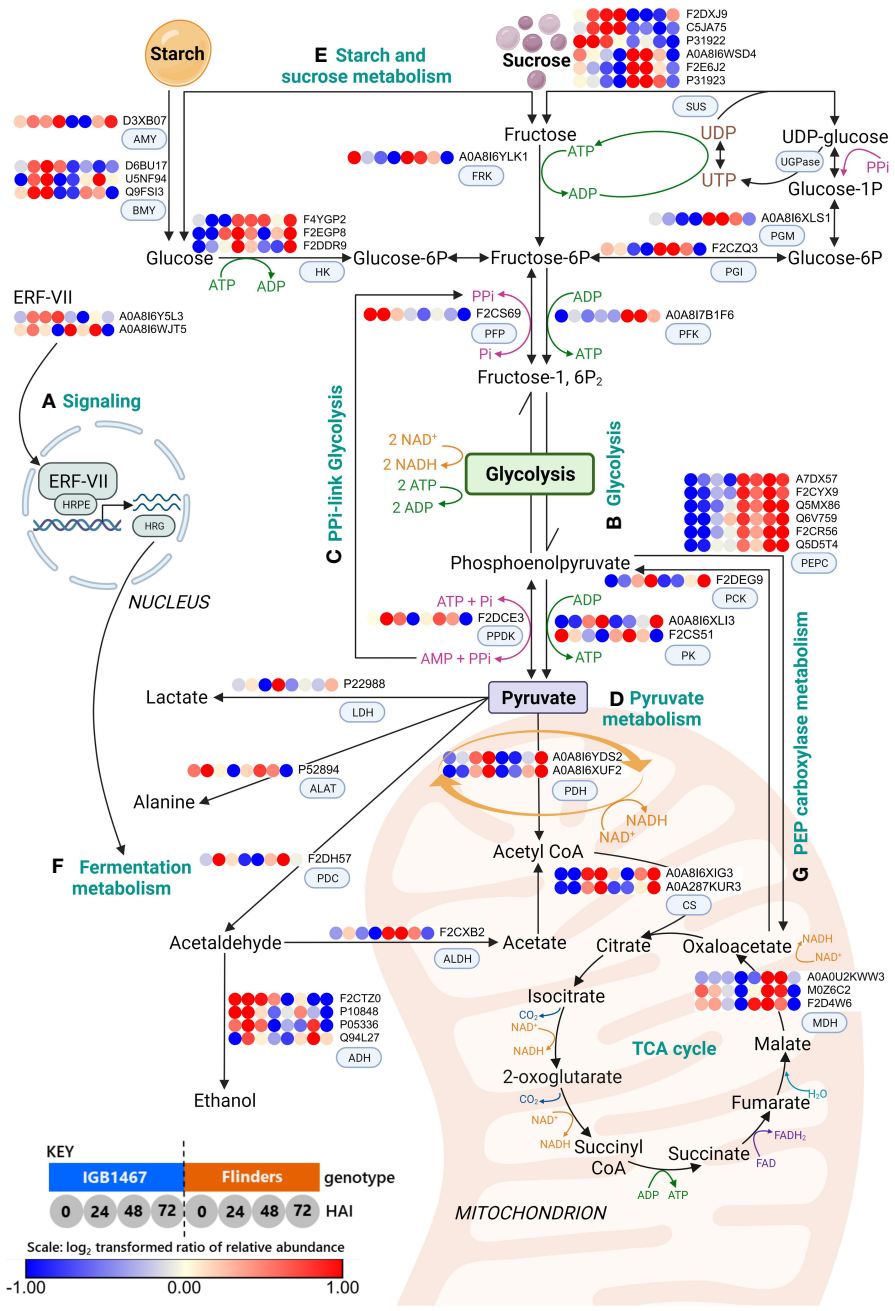
Schematic revealing changes in abundance patterns of DAPs related to signaling, carbohydrate, and fermentation metabolism in malting barley germination: **(A)** Signaling; **(B)** Glycolysis; **(C)** PPi-linked glycolysis; **(D)** Pyruvate metabolism; **(E)** Starch and sucrose metabolism **(F)** Fermentation metabolism; and **(G)** Phosphoenolpyruvate (PEP) carboxylase metabolism. Differentially abundant proteins (DAPs, log_2_ FC≥1.5, FDR≤0.05) were mapped onto the metabolic pathways. The log_2_ protein abundance across the time course of IGB and FLN (n=4, see Materials and Methods) has been plotted into dots as shown in the Key; 1, 2, 3, and 4 represent IGB at 0, 24, 48, and 72 HAI, while dots; 5, 6, 7, 8 represent FLN at 0, 24, 48 and 72 HAI. Scale: Dark blue dots indicate decreased protein abundance, and red dots indicate increased protein abundance (created with BioRender.com). (ADH, alcohol dehydrogenase; AlaAT, alanine aminotransferase; ALDH, aldehyde dehydrogenase; AMY, α-amylase; BMY, β-amylase; CS, citrate synthase; ERF-VII, ethylene response factor; Fructose-1,6P2, fructose 1,6-bisphosphate; Fructose-6P, fructose-6-phosphate; FRK, fructokinase; Glucose-1P, glucose-1-phosphate; Glucose-6P, glucose-6-phosphate; HK, hexokinase; HRPE, hypoxia-responsive promotor element; HRG, hypoxia response gene; LDH, lactate dehydrogenase; PCK, phosphoenolpyruvate carboxykinase; PDC, pyruvate decarboxylase; PDH, pyruvate dehydrogenase; PEPC, phosphoenolpyruvate carboxylase; PEP, phosphoenolpyruvate; PGK, phosphoglycerate kinase; PGM, phosphoglucomutase; PFK, ATP-dependent phosphate dehydrogenase; PFP, PPi-dependent phosphofructokinase; PGI, phosphoglucose isomerase; PK, pyruvate kinase; PPDK, Pyruvate phosphate dikinase; PYR, pyruvate; MDH, malate dehydrogenase; OAA, oxaloacetate; SUS, sucrose synthase; UGPase, UDP-glucose pyrophosphorylase).

To further explore the impacts of low-O_2_ on carbohydrate metabolism we carried out KEGG functional analysis. First, we visualized mitochondrial proteins known to be impacted by low-O_2_ and their influence on energy and signaling proteins (**Supplementary Figure S3**). This was followed by exploration of carbohydrate metabolic pathways, primarily glycolysis, starch and sucrose, and pyruvate metabolism (**Figure 3**). The results of protein groups and abundance patterns involved in these pathways revealed a metabolic rerouting associated with oxygen deficiency, explained further in the discussion.

### Phenotypic compositional verification of malting barley during controlled germination

**Figure 4:**
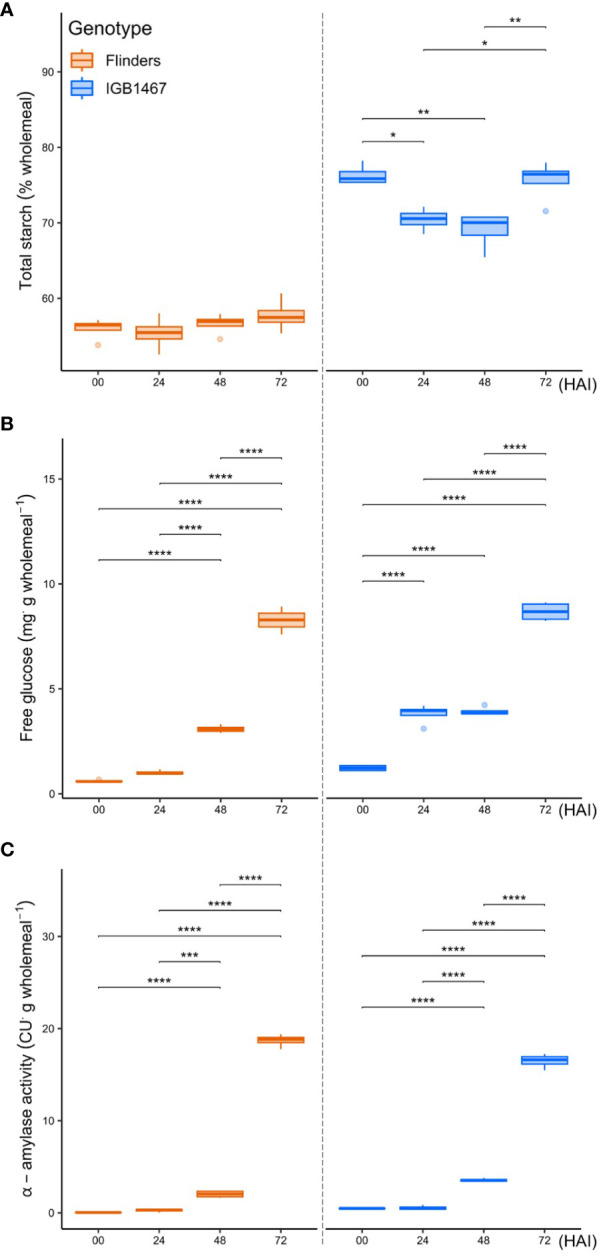
Grain compositional analysis of two malt barley genotypes across four key germination stages (hours after imbibition, HAI). Total starch **(A)** is expressed as a percentage per gram of wholemeal. Free glucose **(B)** is expressed in milligrams per gram wholemeal. Total amylase activity **(C)** is expressed in ceralpha units (CU) per gram wholemeal. The data are presented as the mean ± SD (n = 3 independent technical replicates). Statistical significance was analyzed using one-way ANOVA multiple comparisons followed by a t-test. Asterisks indicate significant differences among the time point (*p<0.05; **p<0.01; ***p<0.001; ****p<0.0001).

Physiochemical analysis (**Figure 4**; **Supplementary Data S4**) showed that the onset of germination significantly impacted the total starch content in IGB but not seen in FLN (**Figure 4A**). In IGB, the largest abundance of starch was seen at 0 and 72 HAI, with a significant drop in starch composition at 24 and 48 HAI, whereas FLN demonstrated no significant changes. There was a significant increase in compositional free glucose of both IGB and FLN across the germination time points (**Figure 4B**). IGB had an earlier onset increase at 0 to 24 HAI (*p*=1.55e-06), not seen in FLN. However, FLN increased from 24 to 48 HAI (*p*=3.17e-06), with no significant change observed in IGB. IGB and FLN had the greatest increase of free glucose from 48 to 72 HAI (*p*=1.49e-09 and *p*=9.20e-11, respectively). Finally, α-amylase activity presented a similar trend for both genotypes, with a non-significant gradual increase from 0-24 HAI and an increase from 24-48 HAI in both IGB and FLN (*p*=1.18e-06, and *p*=2.65e-04, respectively) (**Figure 4C**).

## Discussion

This study explored the proteome and composition of two malting barley genotypes, demonstrating differences in the proteome as measured across controlled germination. The IGB1467 barley genotype demonstrates a more rapid proteome alteration in response to controlled germination with earlier protein abundance changes seen from 24 to 48 HAI, whereas Flinders demonstrates greater changes from 48 to 72 HAI (**Figure 1**; **Supplementary Figure S2**). These findings further contribute to the previously described malting phenotypes and support the hypothesis that IGB1467 demonstrates efficient protein hydrolysis requiring less moisture in contrast to Flinders with slower germination and greater water uptake.

Proteome exploration uncovered several key protein groups responsible for metabolic advantage via alternate modulation between the genotypes in adaptive tolerance to low-O_2,_ where IGB1467 demonstrated less sensitivity than Flinders under low-O_2_ conditions. The steep, during controlled germination, is known to enforce periods of O_2_ deficiency due to submergence regardless of steeping conditions (Wilhelmson et al., 2006). In addition, attempts to aerate the water are inefficient due to the poor solubility of oxygen in water (Kelly and Briggs, 1992). Therefore, we hypothesize that observed differences in IGB1467 and Flinders (**Figures 2**; **3**, **Supplementary Figure S3**) are related to varied metabolic adaptations (‘tolerance’) during controlled germination to low-O_2_ conditions with the regulation of previously reported submergence-responsive pathways (Luan et al., 2018; Borrego-Benjumea et al., 2020; Luan et al., 2022). The observed difference in metabolic modulation of protein groups under low-O_2_ enforced by submergence during controlled germination saw varied patterns between IGB1467 and Flinders related to signaling, protein synthesis, energy metabolism, including glycolysis, fermentation, and finally, the impacts of low-O_2_ metabolic activity and generation of reactive oxygen species. Our results serve as a foundation revealing physiological changes related to water usage of the malting barley grain during the controlled germination process.

### Sensing and signaling low-O_2_ stress is a key adaptive response to controlled germination

In plants, ethylene plays a central role in signaling and modifying responses to low-O_2_ (Bailey-Serres et al., 2012). We found GO terms related to signaling (**Figure 2**, cluster III) that include the ethylene response factor (ERF-VII; APETALA2/Ethylene Response Factor; *AP2/ERF*) and the hypoxia induced protein (HIG1). Under submerged conditions, O_2_ deficiency reduces pyruvate dehydrogenase (PDH) expression, limiting the entry of acetyl-CoA into the mitochondria required for aerobic oxidative phosphorylation (Shingaki-Wells et al., 2014). This leads to an energy crisis and the expression of ERF-VII, known to enhance hypoxia survival in barley (Mendiondo et al., 2016). ERF-VII is a transcription factor that functions as a master regulator of low-O_2_ response, controlled by the cysteine branch of the N-degron pathway (Licausi et al., 2011; van Dongen and Licausi, 2015). During normoxia conditions, ERF-VII is degraded via the ubiquitin-proteasome pathway. However, in hypoxic conditions, ERF-VII is stabilized and protected from proteolysis, allowing for the transcription of hypoxia genes (Fukao et al., 2019; Loreti and Perata, 2020). The ERF-VII transcription factor subfamily is also a master activator of the hypoxia-responsive promotor element (HRPE), which up-regulates a core set of conserved hypoxia-responsive genes (HRG) (Loreti, E., and Perata, 2020; Licausi et al., 2010; Gasch et al., 2015), such as pyruvate decarboxylase (PDC), and alcohol dehydrogenase (ADH) (Mustroph et al., 2010; Gibbs et al., 2011; Giuntoli et al., 2017) discussed in further sections. Both genotypes were responsive to hypoxic conditions with an observed decrease in PDH and mitochondrial respiratory chain protein abundance and an increased abundance of HIG1 and ERF-VII (**Figure 3A**; **Supplementary Figure S3**). IGB1467 demonstrated a greater abundance of ERF-VII earlier than Flinders (**Figure 3**; **Supplementary Figure S3**). This finding suggested that IGB1467 and Flinders both experienced hypoxic conditions enforced by submergence during steeping, sharing similar patterns of HIG1. However, IGB1467 demonstrates earlier low-O_2_ sensing via ERF-VII, possibly contributing to a more responsive and adaptable metabolism. Further studies are required to explore the possible difference signaling cascades involved in the differential, adaptive response mechanism seen in IGB1467 and Flinders.

### IGB1467 demonstrates an earlier onset of reserve mobilization driven by increased protein synthesis

During the controlled germination process, germinating grain lacks photosynthesis and mineral absorption, relying mainly on stored energy and nutrients to maintain normal seed germination. We found several protein GO terms related to storage reserve mobilization (**Figure 2**, cluster I), for example, ‘Macromolecule metabolic process,’ including ‘Proteolysis.’ These terms shared similar abundance patterns between the two genotypes, with low abundance at 0 HAI and increasing over time. However, IGB1467 demonstrates an earlier increase and greater overall abundance. Previous findings on enzymes that aid in the breakdown of these reserves were found to be activated, releasing energy and enzymes, for example, β-amylase, necessary for endosperm modification and embryo growth (Shewry and Halford, 2002; Bewley et al., 2013; Cai et al., 2013; Wang et al., 2015). Our findings confirm an increased abundance of proteins related to storage reserve mobilization, such as proteases, after imbibition and reassumption of metabolism in both genotypes. However, IGB1467 demonstrates a greater abundance of related proteins that may contribute to increased storage reserve mobilization and modification efficiency.

Protein synthesis is crucial to storage reserve mobilization, where enzyme activation and *de novo* synthesis of proteins are required for endosperm modification (Sano et al., 2020). It has been previously reported that mature, dry grain can rapidly restart metabolic activity, including protein synthesis, after imbibition (Bai et al., 2020) by utilizing ‘Long-life mRNAs’ that, upon imbibition, meet the *de novo* protein biosynthesis (He et al., 2011). Our results demonstrated that IGB1467 showed a higher abundance of proteins involved in ‘RNA biosynthesis processes’ and ‘Ribosomal activities’ (**Figure 2**, cluster V), crucial for *de novo* protein synthesis during controlled germination. The significantly higher abundance of these proteins in IGB1467 may allow for a faster adaptive response to imbibition and stress response to low-O_2_ conditions. This leads to elevated protein synthesis and enzyme production to readily mobilize storage reserve during controlled germination. Our findings are supported by earlier studies, suggesting that the synthesis of new proteins and continued gene expression are essential for the acclimation of rice (*Oryza sativa* L.) coleoptiles to low-O_2_ conditions (Huang et al., 2005; Lasanthi-Kudahettige et al., 2007).

### Differentially abundant proteins highlight contrasting adaptive responses to controlled germination

#### Starch and sucrose metabolism feeding glycolysis during controlled germination

During controlled germination, the grain experiences a strong energy and carbon availability decline. Previous studies in rice found some accession can germinate under low-O_2_ conditions but require large reserves of readily available sugars to do so (Magneschi and Perata, 2009; Loreti et al., 2017; Cho et al., 2021). We found that IGB1467 had a greater amount of total starch in the grain, significantly decreasing over the early stages of controlled germination, in contrast to Flinders (**Figure 4A**). To help maintain supply with increased carbon demand under low-O_2_ conditions requires an increase in the conversion of starch to glucose involving amylases, for example, α- and β-amylase, as seen in anoxia-tolerant rice (Lasanthi-Kudahettige et al., 2007). We found greater amylase abundance in IGB1467 (**Figure 3E**), which may have contributed to the increased conversion of starch to glucose, resulting in the earlier increased abundance of free glucose also seen in IGB1467 (**Figure 4B**). However, we did not see a similar increase in compositional α-amylase activity (**Figure 4C**). This suggests that rather than α-amylase, β-amylase activity may contribute to starch degradation and increased free glucose content. Additionally, β-amylase is known to accumulate in the endosperm in both free and bound forms (Ziegler, 1999). During seed germination, bound β-amylase is released in a soluble active form by proteolysis, resulting in a transient increase in total β-amylase activity (Sopanen and Laurière, 1989). Therefore, we can further suggest that the increased proteolysis of IGB1467 contributed to a greater abundance of released β-amylase. Previous studies have shown that greater amylase abundance can be induced in response to low-O_2_ during germination and that expression changes between 24 and 72 HAI positively correlate to embryo growth (Ismail et al., 2009; Hsu and Tung, 2017). This finding suggests that IGB1467, in contrast to Flinders, had greater available free glucose content for glycolysis and energy production via ethanolic fermentation, contributing to the observed increase in modification efficiency.

In addition to starch degradation, SUS is a key enzyme for channeling sucrose into the glycolysis pathway. Under hypoxic conditions, the conversion of sucrose to fructose switches from invertase to SUS, as seen in anoxia-tolerant rice (Lasanthi-Kudahettige et al., 2007; Magneschi and Perata, 2009; Loreti et al., 2017), helping to maintain supply with increased carbon demand (Stark et al., 1992; Kumutha et al., 2008). Both genotypes demonstrated an increased abundance of SUS in the earlier time points (**Figure 4E**). In IGB1467, SUS was present at 24 HAI and increased in abundance over time, whereas Flinders had an increased SUS in the grain (0 HAI) that decreased during controlled germination. In low-O_2_ environments, over-expression of SUS genes in barley conferred tolerance to hypoxic stress (Luan et al., 2022), suggesting the increase in IGB1467 supports greater tolerance to low-O_2_ during controlled germination.

#### Glycolysis and Pyrophosphate-linked glycolysis energy supply during controlled germination

Under hypoxic conditions, the glycolysis pathway is upregulated. We found many proteins involved in catalyzing the conversion of glucose to pyruvate via glycolysis with shared abundance patterns (**Figure 3B**), suggesting both genotypes undertook glycolysis for energy production, reported previously in barley (Borrego-Benjumea et al., 2020). Moreover, these results are similar to findings in canola (*Brassica napus* L.) under waterlogging stress, with both tolerant and sensitive varieties showing high induction of glycolysis-related genes (Zou et al., 2015). This suggests that glycolysis is a shared response irrespective of tolerance to submergence during controlled germination (Zou et al., 2015). Therefore, the abundance of these proteins was not seen as a key contributor to low-O_2_ tolerance between genotypes.

In addition to glycolysis, pyrophosphate-dependent phosphofructokinase (PPi-PFK) and pyruvate orthophosphate dikinase (PPDK) proteins demonstrated varied abundance in IGB1467 and Flinders. In IGB1467, we saw a significant increase in PPi-PFK and PPDK (**Figure 3C**). Previous studies in rice found that low-O_2_ conditions led to increased expression of PPi-PFK and PPDK and are involved in PPi-linked glycolysis as an alternative energy currency (Huang et al., 2005; Lasanthi-Kudahettige et al., 2007; Atwell et al., 2015; Hsu and Tung, 2017). PPi-PFK plays a role in conserving ATP levels by utilizing PPi generated by PPDK instead of ATP-dependent 6-phosphofructokinase (ATP-PFK) in the glycolytic pathway, conserving ATP (Gibbs and Greenway, 2003; Ferjani et al., 2011; Igamberdiev and Kleczkowski, 2021). Our findings suggest IGB1467 switched to PPi-linked glycolysis, substituting PPi-PFK for ATP-PFK during glycolysis and increasing ATP yield under low-O_2_ conditions. In rice, PPi utilization alleviated the anoxia ATP deficiency to preserve cellular processes, including protein synthesis (Huang et al., 2005). Therefore, we hypothesize this alternate energy source may provide IGB1467 flexibility not seen in Flinders to preserve energy, maintain cellular processes, and support previously discussed protein synthesis for continued grain modification under low-O_2_ conditions during controlled germination.

In Flinders, despite the observed increased abundance of PPDK, there was a low abundance of PPi-PFK and a higher abundance of ATP-PFK, suggesting the PPi is used in an alternate pathway to support sucrose (Suc) conversion of UDP-glucose to glucose-1-phosphate (Glc-1-P), which can then be directed toward glycolysis (Atwell et al., 2015). Our results support this hypothesis with Flinders demonstrating an increased abundance of subsequent enzymes, phosphoglucomutase (PGM) and phosphoglucose isomerase (PGI), involved in feeding fructose into the glycolysis pathway (**Figure 3**). Therefore, it has been demonstrated that PPi can generate equilibrium changes to bypass ATP-dependent metabolism, providing plasticity in the metabolism during the energy crisis of low-O_2_ stress during controlled germination (Igamberdiev and Kleczkowski, 2021).

#### Rerouting of energy metabolism via pyruvate during controlled germination

The final product of glycolysis is NADH and pyruvate, which are transferred to the TCA cycle via PDH under normoxic conditions to create more ATP. However, under controlled germination conditions, we saw a decrease in mitochondrial PDH in both IGB1467 and Flinders (**Figure 3D**; **Supplementary Figure S3**). As discussed earlier, PDH plays an important role in the conversion of pyruvate to acetyl-CoA for entry into the TCA, therefore limiting carbon entering the TCA cycle and re-routing energy metabolism (Bailey-Serres and Voesenek, 2008; Zhang et al., 2023). This finding suggests that both genotypes experienced hypoxic conditions and that adaptive flexibility within the controlled germination pivots on the pyruvate metabolism.

The pyruvate metabolism under low-O_2_ involves three pathways: (1) alanine synthesis, (2) lactic acid fermentation, and (3) ethanol fermentation (**Figure 3F**). While these three pathways utilize pyruvate as a substrate, ethanolic and lactic acid fermentation regenerate NAD^+^ via PDC and lactate dehydrogenase (LDH). Whereas alanine synthesis via alanine aminotransferase (AlaAT) stores carbon and nitrogen (Ricoult et al., 2005). We found several proteins in the pyruvate metabolism. LDH was low in IGB1467 and Flinders in the early stages of controlled germination, with a greater abundance of PDC and AlaAT, respectively. This finding suggests that in both genotypes, pyruvate was diverted away from lactic acid fermentation, and different genotype-specific re-rerouting strategies were employed under low-O_2_ stress of controlled germination.

AlaAT is responsible for increased alanine synthesis detected in both genotypes at 24 HAI with greater abundance in IGB1467. However, abundance in IGB1467 decreased from 24 to 48 HAI, not seen in Flinders (**Figure 3F**). This suggests increased carbon/nitrogen storage in Flinders (Diab and Limami, 2016). Ricoult, Cliquet (Ricoult et al., 2005) found in Medicago (*Medicago truncatula*), AlaAT expression increased under anoxic conditions, competing with ethanolic fermentation for pyruvate to increase alanine synthesis. Alanine accumulation contributed to anoxia tolerance, saving carbon as stored nitrogen and limiting the accumulation of the toxic compound acetaldehyde (Ricoult et al., 2005). In addition, during post-hypoxia recovery, stored carbon can be mobilized to produce pyruvate via the reverse reaction of AlaAT/glutamate dehydrogenase and funneled into the TCA cycle (Diab and Limami, 2016). This finding suggests that in contrast to IGB1467, Flinders rerouted pyruvate to alanine instead of ethanolic fermentation. In addition, this strategy may aid in energy production upon the reassumption of aerobic respiration during germination. Overall, our data support the hypothesis that genotype-specific re-routing strategies in the pyruvate metabolism are key to sensing low-O_2_ and facilitating adaptability in response to low-O_2_ conditions during controlled germination.

IGB1467 demonstrated ethanol fermentation with a greater abundance of PDC involved in turning pyruvate into acetaldehyde, which is then reduced to ethanol by ADH (Ismond et al., 2003; Shingaki-Wells et al., 2014; Ventura et al., 2020). These enzymes are among the ‘core hypoxia response’ genes (Lee et al., 2014; Mustroph et al., 2014) known to be essential for submergence tolerance in barley under hypoxic conditions caused by waterlogging stress (Zhang et al., 2016; Zhang et al., 2017; Luan et al., 2018; Luan et al., 2022). Our study found that ADH had greater abundance in IGB1467 compared to Flinders (**Figure 3F**). The increased abundance of PDC at 24 HAI may have contributed to the increased abundance of ADH also seen at 24 and 48 HAI in IGB1467. The observation that IGB1467 had a greater abundance and earlier onset of essential anaerobic proteins PDC and ADH suggests switching to the fermentation metabolism to maintain energy via glycolysis. Additionally, we can hypothesize that this alternative energy supply could further support greater protein synthesis and increased protein hydrolysis observed in IGB1467 seen to a lesser extent in Flinders.

In contrast to fermentation, Flinders demonstrated differential modulation of pyruvate metabolism, as seen in the significantly increased abundance of phosphoenolpyruvate carboxylase (PEPC) not seen in IGB1467 (**Figure 3G**). PEPC is at the core of plant carbon fixation, assimilating CO_2_ during crassulacean acid metabolism (CAM) photosynthesis (O'Leary et al., 2011). However, PEPC also plays a role in several non-photosynthetic functions. For example, when coupled with malate dehydrogenase (MDH) and NAD^+^-dependent malic enzyme (NAD-ME), it forms an alternative flux that can function in place of pyruvate kinase (PK) to generate pyruvate (Sweetlove et al., 2010; O'Leary et al., 2011) as seen in Flinders. However, previous findings in rice have found a strong suppression of PEPC in hypoxic-tolerant lines compared to a low-level reduction in hypoxia-sensitive lines (Lasanthi-Kudahettige et al., 2007; Hsu and Tung, 2017). Hsu and Tung (2017) hypothesized that because PEPC converts PEP into oxaloacetate rather than pyruvate, there is insufficient pyruvate for ethanol production, bypassing alcohol fermentation and contributing to increased sensitivity to low-O_2_ conditions (Hsu and Tung, 2017). This redirection of pyruvate was evident in Flinders, where we saw a significantly lower abundance of fermentation-related proteins than IGB1467. This finding further supports Flinders’s sensitivity to low-O_2,_ where previous studies have demonstrated that the abundance of enzymes participating in fermentative ethanol production involving PDC and ADH are significantly higher in ‘hypoxia tolerant’ germinating rice seeds compared to ‘hypoxia sensitive’ lines (Ismail et al., 2009; Miro et al., 2017). Therefore, this suggests impaired ethanol production in Flinders leads to hypoxic sensitivity due to insufficient energy generation for embryo growth via transcriptional regulation (Hsu and Tung, 2017).

### Impacts of rerouting for efficient modification under controlled germination

Submergence and low-O_2_ stress experienced during controlled germination leads to the accumulation of reactive oxygen species (ROS). Under oxygen deficiency, disruption of the electron transport chain in mitochondria results in excess hydrogen peroxide (H_2_O_2_), and increased ROS generation causing cell oxidative damage (Fukao and Bailey-Serres, 2004; Shabala et al., 2014; Jacoby et al., 2018). As seen in previous studies of barley under waterlogging conditions and in the early stages of malting (Gill et al., 2019; Mahalingam et al., 2021). However, an effective antioxidant metabolism can alleviate the harmful impacts of ROS (Apel and Hirt, 2004; Mittler, 2017) aided by several antioxidant enzymes, for example, superoxide dismutase (SOD), peroxidase (POD), ascorbate peroxide (APX) and catalase (CAT) (Blokhina et al., 2003). In our study, 32 proteins involved in ‘Cellular oxidant detoxification’ activity were found with contrasting abundance patterns between the two genotypes (**Figure 2**, cluster II), IGB1467 having low abundance and Flinders having significantly greater abundance. The proteins involved in this cluster included APX and POD, most of which were related to peroxidases (24 proteins, 85% of 28). Luan, Guo (Luan et al., 2020) found that over-expression of barley *HvERF2.11* in Arabidopsis (*Arabidopsis thaliana*) triggered the increased expression level of antioxidant enzyme biosynthesis genes (*AtSOD1*, *AtPOD1*) and ethylene biosynthesis gene (*AtACO1*). They conferred resistance to waterlogging stress (Luan et al., 2020). This suggests that although IGB1467 demonstrates greater tolerance to low-O_2_ conditions, the grain may not have the protective functions that exist in Flinders for ROS detoxification, which may have an undesirable impact on malt quality. However, this will require further investigation to confirm the ROS production and specific effects of antioxidant enzyme activities and their influence on malt quality.

## Conclusion

In this study, we explored the proteomes of two malting barley genotypes with different malting phenotypes during controlled germination. In contrast to Flinders, we identified differences in protein abundance patterns that suggest IGB1467 has better adaptability or ‘less sensitivity’ to low-O_2_ conditions experienced during submergence of controlled germination, contributing to more efficient endosperm protein hydrolysis at lower grain moisture. Although the treatments were varied, it was evident that both genotypes experienced low-O_2_ conditions with an increase in ethylene and hypoxic response signaling. The stress induced by this O_2_ deficiency led to various metabolic adaptations and rerouting carbohydrate metabolism, where the continued efficient proteolysis in IGB1467 may be attributed to increased protein synthesis (seen in the mature grain) and a switch to fermentation and PPi-linked energy pathways to support the boosted protein synthesis and overall modification. However, the greater low-O_2_ tolerance seen in IGB1467 is likely a result of several interaction factors at the molecular, biochemical, and anatomical levels. Further studies are required to understand the ethylene signaling pathway and the potential impacts of reduced antioxidant activity seen in IGB1467 on malt quality. Our findings shed light on the consequence submergence during controlled germination, providing insight into mechanisms for low-O_2_ sensing in barley grain, the underestimated role of the fermentative metabolism in efficient grain modification, highlighting possible adaptive metabolic traits as breeding targets for optimizing malting practices.

## Data availability statement

The datasets presented in this study can be found in online repositories. The names of the repository/repositories and accession number(s) can be found below: CSIRO Data Access Portal Accession id: https://doi.org/10.25919/ypsj-4d27.

## Author contributions

CO’L: Conceptualization, Data curation, Formal Analysis, Investigation, Methodology, Validation, Visualization, Writing – original draft. AJ: Conceptualization, Methodology, Resources, Supervision, Writing – review & editing. MN-W: Methodology, Resources, Supervision, Validation, Writing – review & editing. HD: Conceptualization, Methodology, Resources, Supervision, Writing – review & editing. DM: Conceptualization, Funding acquisition, Resources, Supervision, Writing – review & editing. J-PR: Conceptualization, Funding acquisition, Methodology, Resources, Supervision, Writing – review & editing. MC: Conceptualization, Funding acquisition, Methodology, Project administration, Resources, Supervision, Writing – review & editing.

## Footnotes

1.
https://www.ipk-gatersleben.de/

2.
http://eggnog-mapper.embl.de/ (Cantalapiedra et al., 2021)

3.
http://geneontology.org/docs/go-enrichment-analysis/ (GO-Consortium, 2021)

4.
https://www.uniprot.org/id-mapping (UniProt-Consortium, 2022)

5.
https://ics.hutton.ac.uk/eorna/index.html (Rapazote-Flores et al., 2019)

6.
https://ics.hutton.ac.uk/eorna/download.html

7.
https://www.kegg.jp/kegg/pathway.html (Kanehisa et al., 2023)

8.
http://bioinformatics.sdstate.edu/idep96/ (Ge et al., 2018)

9.
https://software.broadinstitute.org/morpheus/

10.
https://www.biorender.com/

11.
https://www.r-project.org/

12.
https://rstudio.com
