# Supplementary material for: Proteomic exploration reveals a metabolic rerouting due to low oxygen during controlled germination of malting barley (Hordeum vulgare L.)
Source: Front Plant Sci. 2023 Dec 11;14:1305381. doi: 10.3389/fpls.2023.1305381 (PMC10771735; doi:10.3389/fpls.2023.1305381)
Supplement: Supplementary file 1 [file DataSheet_1.docx]

Supplementary Material

## Supplementary Figures


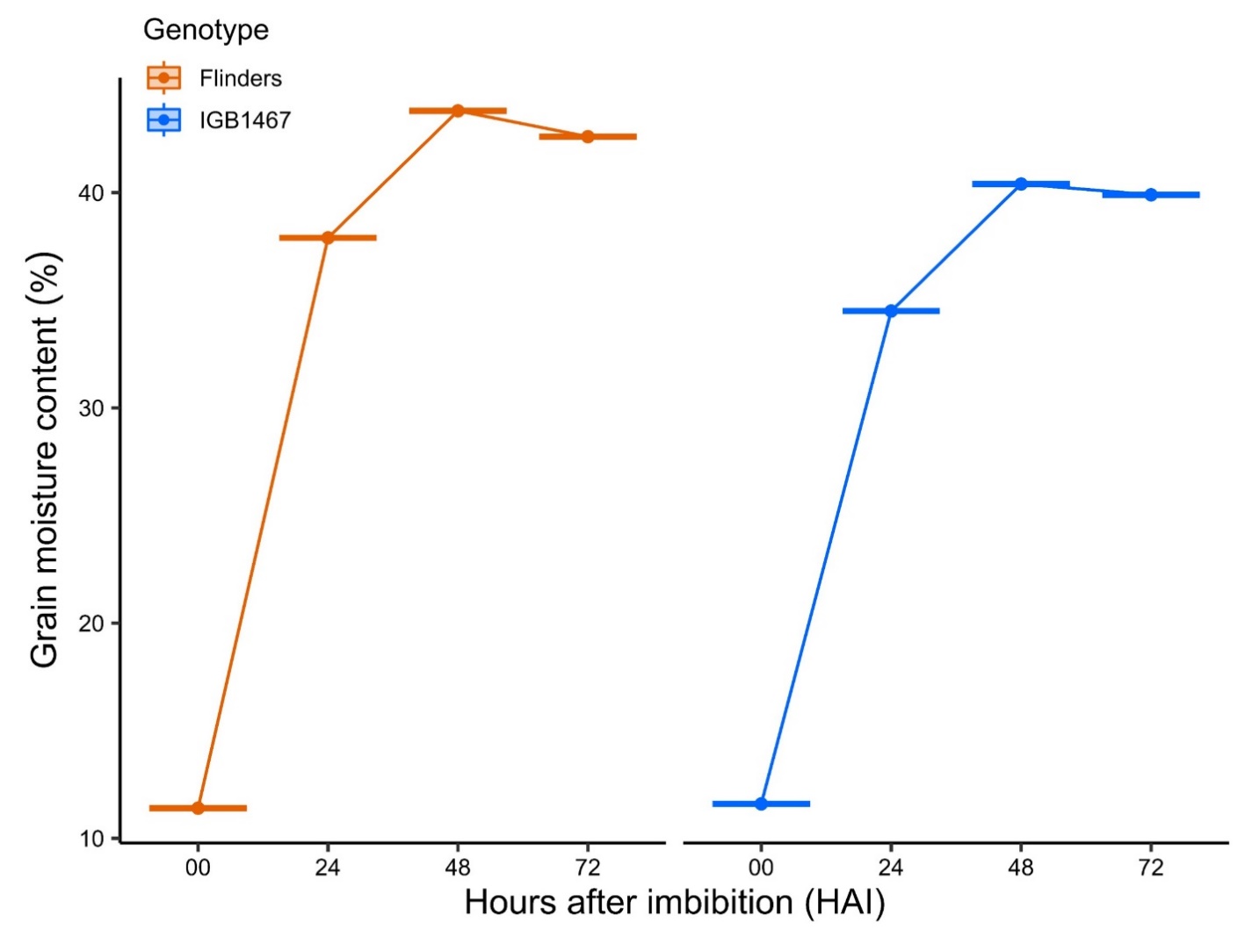


SUPPLEMENTARY FIGURE S1. Grain moisture content of IGB1467 and Flinders at 0, 24, 48, and 75 hours after imbibition during controlled germination (HAI) (courtesy of Pilot Malting Australia).


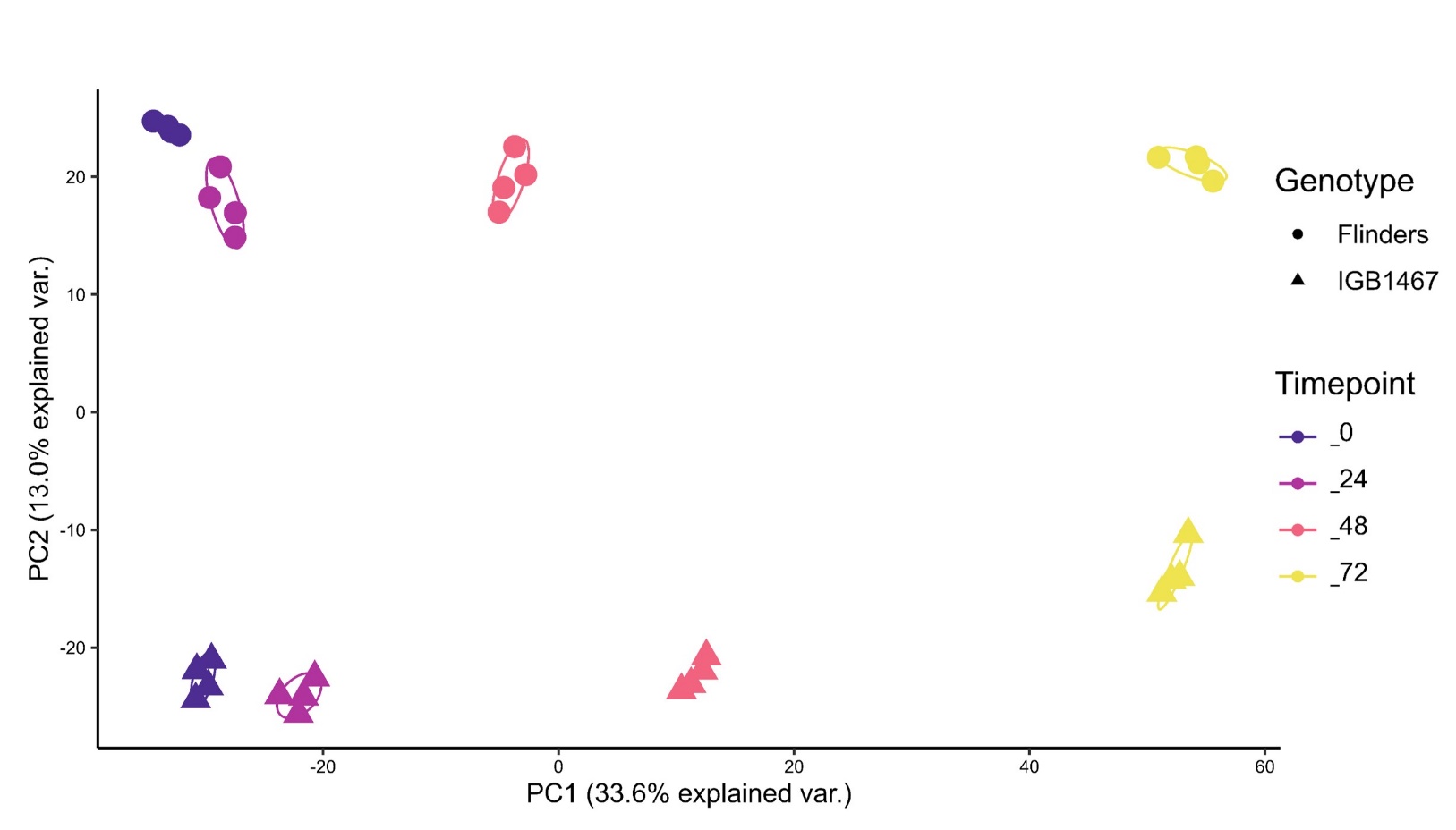


SUPPLEMENTARY FIGURE S2**.** Unsupervised principal component analysis of data variance between Flinders and IGB1467 at 0, 24, 48, and 72 HAI.


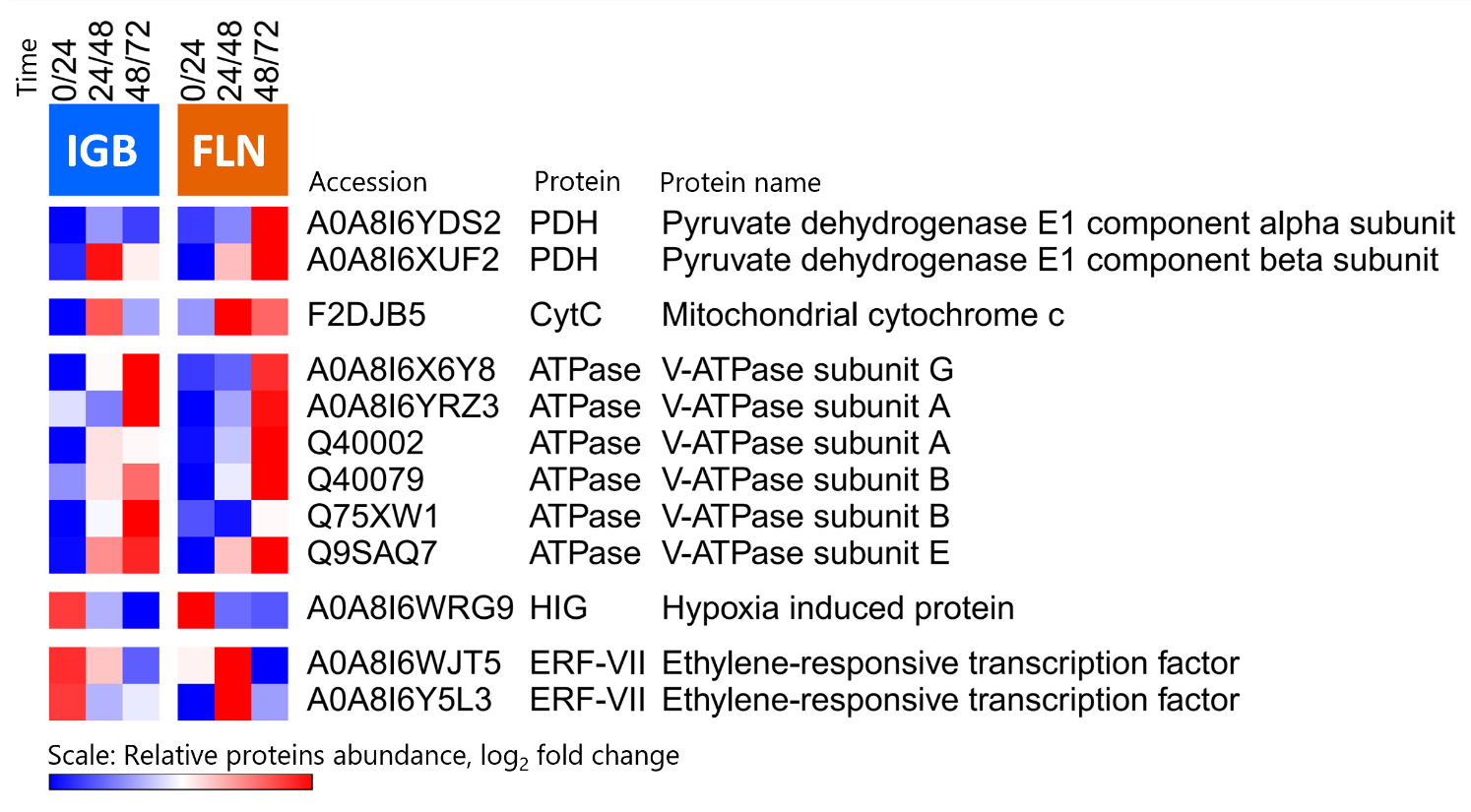


SUPPLEMENTARY FIGURE S3. Heatmap revealing relative protein abundance patterns (log_2_ fold change) change across the time course of IGB and FLN (n =4) related to energy production and signaling under submergence during controlled germination. Timepoint comparisons include 0/24, 24/48, and 48/72 HAI. Scale: Blue indicates decreased protein abundance, and red indicates increased protein abundance.


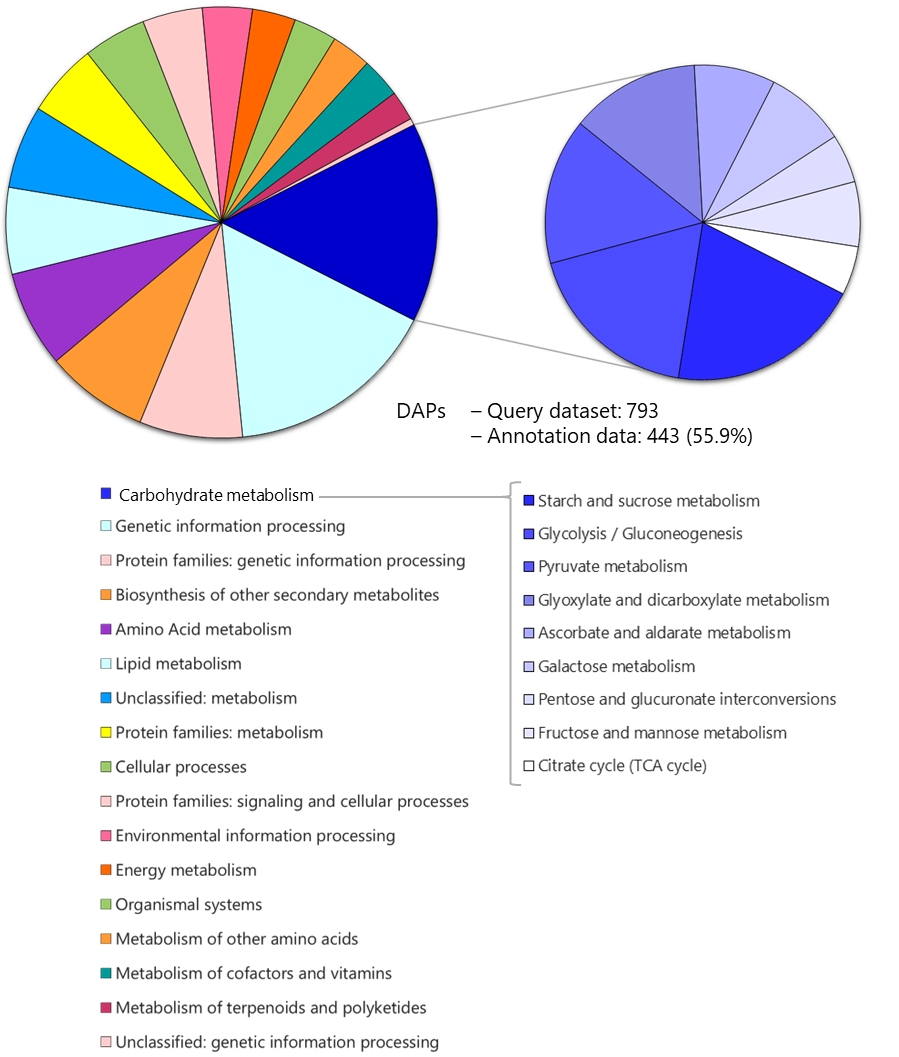


SUPPLEMENTARY FIGURE S4. Pie of pie plot of functional classification, KEGG ortholog (KO) for all differentially abundant proteins found in IGB and FLN and their KEGG pathways, BRITE hierarchies, and KEGG modules. KEGG annotation query dataset of 793 443 entries annotated (55.9%). Pie 1 is the functional classification highlighted in the key, and Pie 2 is the breakdown of carbohydrate functional classification.

## Supplementary Tables

SUPPLEMENTARY TABLE S1. Quality of grain samples (courtesy of Barrett Burston Malting)

|  | | | |  | **SCREENINGS (%)** | | | |
| --- | --- | --- | --- | --- | --- | --- | --- | --- |
| Sample | RVA | %M | Grain protein content (%) | Total nitrogen | >2.8 | >2.5 | >2.2 | <2.2 |
| IGB1467 | 148 | 11.6 | 9.8 | 1.57 | 73 | 11.7 | 2.4 | 0.1 |
| Flinders | 171 | 11.4 | 9.6 | 1.54 | 59.4 | 35.6 | 4.9 | 0.1 |

SUPPLEMENTARY TABLE S2. Pilot Malting Australia malting batch details (courtesy of Pilot Malting Australia)

|  |  |  | **1^st^ Steep** | | **Rest** | | **2^nd^ Steep** | | **Total Steep** | **1^st^ Germination** | | **2^nd^ Germination** | | **Total Germination** | **Initial Kiln *controlled by breakthrough** | | **Final Kiln** | | **Total** | **Total malting time** |
| --- | --- | --- | --- | --- | --- | --- | --- | --- | --- | --- | --- | --- | --- | --- | --- | --- | --- | --- | --- | --- |
| Sample | PMA run | Grain protein content (%) | (h) | (^°^C) | (h) | (^°^C) | (h) | (^°^C) | (h) | (h) | (^°^C) | (h) | (^°^C) | (h) | (h) | (^°^C) | (h) | (^°^C) | (h) | (h) |
| IGB1467 | 0219 | 9.8 | 8 | 20 | 10 | 20 | - | - | 18:00 | 24 | 18 | 72 | 15 | 96:00 | 7:15 | 60 | 11:10 | 82 | 19:05 | 133:05 |
| Flinders | 0319 | 9.6 | 8 | 20 | 10 | 20 | 4 | 20 | 22:00 | 72 | 16 | 24 | 15 | 96:00 | 7 | 60 | 11:10 | 82 | 18:50 | 136:50 |

SUPPLEMENTARY TABLE S3. Pilot Malting Australia malting batch treatment details (courtesy of Pilot Malting Australia)

|  |  |  | **Water: Seed** | | **Water additions** | | | | | | | | **Air agitation** | | | |
| --- | --- | --- | --- | --- | --- | --- | --- | --- | --- | --- | --- | --- | --- | --- | --- | --- |
|  |  |  | **1^st^ Wet** | **2^nd^ Wet** | **0 hrs** | | **3 hrs** | | **6 hrs** | | **12 hrs** | | **1^st^ Wet** | | **2^nd^ Wet** | |
| Sample | PMA batch | Grain protein content (%) | Ratio | Ratio | (L) | (min) | (L) | (min) | (L) | (min) | (L) | (min) | Duration (min) | Interval (min) | Duration (min) | Interval (min) |
| IGB1467 | 0219 | 9.8 | 6:1 | 5:1 | 7.50 | 5 | 7.5 | 5 | - | - | - | - | 2 | 30 | 5 | 30 |
| Flinders | 0319 | 9.6 | 6:1 | 5:1 | 7.50 | 5 | 7.5 | 5 | 7.50 | 5 | 7.5 | 5 | 2 | 30 | 5 | 30 |

SUPPLEMENTARY TABLE S4. Malt quality analyses after pilot malting of IGB1467 and Flinders (courtesy of Barrett Burston Malting)

| Sample | PMA batch | Peak germ. moisture (%) | Malt moisture (%) | Soluble protein (%) | Kolbach index (%) | FG Extract (%) | Wort Colour (EBC) | Viscosity (mPa s) | Diastatic Power (WK) | AAL (%) | FAN (mg/L) | Friability (%) | WBG (mg/L) | DMSp (mg/kg) |
| --- | --- | --- | --- | --- | --- | --- | --- | --- | --- | --- | --- | --- | --- | --- |
| *Target specifications* | | | *5.0 max* |  | *37-47* | *81.0 min.* | *3.0-4.5* | *1.56 max.* | *300 min.* | *81-86* | *150 min.* | *80.0 min.* | *150 max.* | *5.0 max.* |
| IGB1467 | 0219 | 40.6 | 4.7 | 4.37 | 45 | 82.3 | 3.3 | 1.60 | 258 | 81.9 | 176 | 84.8 | 309 | 3.2 |
| Flinders | 0319 | 43.8 | 4.4 | 4.13 | 43 | 82.5 | 3.0 | 1.51 | 314 | 83.5 | 183 | 94.8 | 62 | 4.3 |

SUPPLEMENTARY TABLE S5. Cluster accumulation pattern results from the PGSEA with HCA of the top 50 significant gene ontology (GO) enriched biological process pathways.

| Cluster I (Figure 3, light blue) displays a low abundance at 0 to 24 HAI, a significant increase from 24 to 48, and a further increase from 48 to 72 HAI. The cluster is primarily enriched in proteins involved in the ‘Carbohydrate metabolic process’ (*p*=3.5e-22). Although both genotypes shared a similar pattern, IGB demonstrated a more rapid increase in abundance, with many changes occurring by 48 HAI but seen later in FLN at 72 HAI. The most significant BP-enriched GO term is ‘Cellular macromolecule catabolic process’ (*p*=5.2e-19), with the largest number of DAPs found in ‘Catabolic process’ (*p*=4.2e-15) and ‘Proteolysis’ (*p*=3.8e-17). |
| --- |
| Cluster II (Figure 3, dark blue) has a significantly greater number of DAPs present in FLN than IGB at all time points, with the largest abundance seen at 48 HAI. The significant GO terms include ‘Response to oxidative stress’ (*p*=2.5e-14) and ‘Hydrogen peroxide catabolic process’ (*p*=5.7e-13), with the largest number of proteins found in ‘Cellular response to chemical stimulus’ (*p*=2.5e-14) and ‘Cellular oxidant detoxification’ (*p*=1.2e-13). Closely related within the clusters with an earlier abundance seen in FLN at 0, 24, and 48 HAI, yet not seen in IGB, was related to transport, ‘Transmembrane transport’ (*p*=1.1e-12). This finding suggests that FLN demonstrates a proteome responsive to stress and cellular detoxification, aided by the increased abundance of transmembrane transport found in FLN. |
| Clusters III, IV, and VI (Figure 3, light green, dark green, and red) vary in abundance patterns between the genotypes, but they share a general trend with the greatest abundance in the raw grain and decreasing across the time course. Cluster III is enriched with ‘Cell communication’ (*p*=2.2e-14), Cluster IV is mainly related to the ‘Response to stimulus’ (*p*=5.7e-18), and Cluster VI ‘Response to biological process’ (*p*=5.0e-13), including ‘Protein modification’ (*p*=13.e-17). |
| Clusters V (Figure 3, pink) displayed a dissimilar pattern between the genotypes, with the greatest abundance observed in IGB at 0 and 24 HAI, gradually decreasing over the remaining time course, not seen in FLN. The most significant GO pathways with the largest number of proteins involve ‘Gene expression’ (*p*=1.4-17) and include ‘Translation’ (*p*=1.6e-16), ‘Peptide biosynthetic process’ (*p*=2.5e-16), and ‘RNA metabolic process’ (*p*=8.1e-15), all linked to protein synthesis. Closely related, another large number of DAPs was found in ‘Regulation of biological process’ (*p*=9.2e-13) followed by the daughter term ‘Regulation of metabolic process’ (*p*=9.2e-13). This cluster is involved in DAPs regulating metabolic processes via protein synthesis, suggesting this is a major contributing factor to IGB's rapid metabolic response at the onset of controlled germination. |
| Overall, there are four distinct clusters (III, IV, V, and VI) corresponding to the first two stages (0 and 24 HAI) and one distinct cluster (I) to the last two stages (48 and 72 HAI). One cluster (II) was found only in FLN with increased abundance across the first three points (0, 24, and 48 HAI). DAP abundance varied most significantly amongst Clusters I, II, and V. This suggests that the proteome response at the onset of controlled germination differed between the two genotypes. |
